# Detection of SARS-CoV-2 in exhaled breath from non-hospitalized COVID-19-infected individuals

**DOI:** 10.1038/s41598-022-15243-1

**Published:** 2022-07-01

**Authors:** Cæcilie Leding, Julia Skov, Katrine Uhrbrand, Jan Gorm Lisby, Katrine Pedersbæk Hansen, Thomas Benfield, Louise Katrine Duncan

**Affiliations:** 1 Department of Infectious Diseases, Center of Research and Disruption of Infectious Diseases, Copenhagen University Hospital-Amager and Hvidovre, Hvidovre, Denmark; 2 AeroCollect A/S, Brøndby, Denmark; 3 Department of Human Diagnostics, FORCE Technology, Brøndby, Denmark; 4 Department of Clinical Microbiology, Copenhagen University Hospital-Amager and Hvidovre, Hvidovre, Denmark; 5 Department of Clinical Medicine, Faculty of Health and Medical Sciences, University of Copenhagen, Copenhagen, Denmark

**Keywords:** Infectious diseases, Infectious-disease diagnostics

## Abstract

The diagnosis of COVID-19 is based on detection of SARS-CoV-2 in oro-/nasopharyngel swabs, but due to discomfort and minor risk during the swab procedure, detection of SARS-CoV-2 has been investigated in other biological matrixes. In this proof-of-concept study, individuals with confirmed SARS-CoV-2 infection performed a daily air sample for five days. Air samples were obtained through a non-invasive electrostatic air sampler. Detection of SARS-CoV-2 RNA was determined with qRT-PCR. The association of positive samples with different exposures was evaluated through mixed-effect models. We obtained 665 air samples from 111 included participants with confirmed SARS-CoV-2 infection. Overall, 52 individuals (46.8%) had at least one positive air sample, and 129 (19.4%) air samples were positive for SARS-CoV-2. Participants with symptoms or a symptom duration ≤ four days had significantly higher odds of having a positive air sample. Cycle threshold values were significantly lower in samples obtained ≤ 4 days from symptom onset. Neither variant of SARS-CoV-2 nor method of air sampling were associated with a positive air sample. We demonstrate that SARS-CoV-2 is detectable in human breath by electrostatic air sampling with the highest detection rate closest to symptom onset. We suggest further evaluation of the air sampling technique to increase sensitivity.

## Introduction

The gold standard in the diagnosis of COVID-19 is detection of SARS-CoV-2 genomes from naso- or oropharyngeal (OP) swabs using real-time reverse transcription polymerase chain reaction (RT-PCR). However, the procedure of obtaining material from the upper respiratory tract is invasive and thus uncomfortable, as well as posing a minor risk for the tested individual^1,2^. Furthermore, it requires trained staff that risk infection following virus exposure during the swabbing procedure. Therefore, detection of SARS-CoV-2 based on other sample materials have been investigated including plasma, serum, urine, and feces^3^. It is well-known that the virus, among other routes, transmits through aerosols and droplets from infected individuals. This has given the rationale of investigating exhaled breath as an alternative specimen type in both the diagnosis of COVID-19 as well as a focus on the strategy to mitigate the transmission of SARS-CoV-2^4–6^. Air sampling of exhaled breath could provide an easy non-invasive testing alternative with potential for self-sampling. Furthermore, studying the emission of SARS-CoV-2 in exhaled breath of infected individuals can provide valuable information e.g. in relation to identifying high risk exposure activities.

A previous study established that SARS-CoV-2 was detectable in air close to the snouts of minks using an electrostatic air sampler (AeroCollect, FORCE Technology, Hørsholm, Denmark)^7^. The aim of the current proof-of-concept study was to investigate whether the AeroCollect device was capable of detecting SARS-CoV-2 in exhaled breath from humans positive for SARS-CoV-2 verified by RT-PCR by OP swab. Additionally, the effect of the variant of SARS-CoV-2, the duration of symptoms and method of air sampling on the detection rate were investigated.

## Material and methods

### Study cohorts

This prospective, observational proof-of-concept study was performed at Amager-Hvidovre Hospital, Copenhagen, Denmark, between December 2020 and May 2021.

Individuals with RT-PCR confirmed SARS-CoV-2 infection up to four days prior to inclusion were identified through the Department of Clinical Microbiology. Eligible participants (not hospitalized and ≥ 18 years) and their household members (not hospitalized and ≥ 12 years) were invited to participate in the study. Prior to study entry, all participants provided written informed consent. The study was approved by the Regional Data Protection Centre (record no. P-2020-1219). The study was exempt from ethical approval according to the Regional Ethics Committee of the Capital Region of Denmark (record no. H-20020554). This decision was made because the committee assessed that the aim of the current study was to achieve knowledge about SARS-CoV-2 in the environment and not about human biology. All methods were performed in accordance with the relevant guidelines and regulations.

Oropharyngeal swabs for RT-PCR was obtained from household members at day one and five. The swabs were analyzed at the Department of Clinical Microbiology and sequenced if SARS-CoV-2 was detected. Clinical information at baseline from each participant and information about symptoms of SARS-CoV-2 infection was provided in a questionnaire from day one to five and registered in the REDCap database.

### Sample collection

Sampling of aerosolized SARS-CoV-2 was carried out using the hand-held AeroCollect air sampling technology (Supplementary Fig. S1). The AeroCollect air sampler was mounted with a chip-based sample chamber and operated at a flowrate of 0.2 L per minute. All samples were self-sampled by the participants, after a brief instruction on how to use the AeroCollect. During sampling, air is pulled into the sample chamber while high voltage generates an electrostatic field across the chamber. The electrostatic field captures and lyses virus (Supplementary Fig. S1). Particle emission rates of aerosols have been shown to be positively correlated with vocal loudness, and thus are higher while singing and loud talking compared to normal talking or breathing^8–10^. Therefore, air samples in this study were obtained from the breathing zone of the participants while singing or talking loudly.

Participants were requested to provide one air sample per day during five consecutive days. The air sampling was performed for four minutes per sample. A subset of participants were asked to provide two matched air samples a day for five consecutive days, one singing/loud talking for four minutes and one exhaling air for four minutes, in order to evaluate different methods of air sampling. This subgroup consisted of all enrolled participant during a specific time period.

All samples were blind coded and transported within one week of last sampling day to FORCE Technology, Hørsholm, Denmark for analysis.

### Detection of SARS-CoV-2 in air samples

Aerosolized SARS-CoV-2 were eluted from the AeroCollect sample chamber using 25 µl nuclease-free water, flushing the chamber 15 times (Supplementary Fig. S1). The sample is ready for RT-PCR without any prior purification steps. The Agilent AriaMX Real-time PCR system (Agilent, United States) was used for detection of SARS-CoV-2. The detection was performed in duplicates using the ViroType SARS-COV-2 detection assay, consisting of the Virotype Mix + IC(JOE)-RNA reaction mixture (INDICAL BIOSCIENCE, Germany) and the Virotype SARS-CoV-2 (E-Sarbecco) Primers/Probes mix (INDICAL BIOSCIENCE, Germany), targeting the E gene of SARS-CoV-2. The RT-PCR reaction was carried out according the manufacturer’s recommendations using eight µl sample template in a total reaction volume of 25 µl. Reaction conditions were RT at 50 °C for 10 min, activation at 95 °C for two minutes followed by 45 cycles of 95 °C for five seconds and 60 °C for 30 s.

All reactions contained an internal reaction control. In each RT-PCR run, a positive control consisting of the Virotype SARS-CoV-2 (E-Sarbecco) Positive Control (INDICAL BIOSCIENCE, Germany) and a negative template control (nuclease-free water) was included. Fluorescence was measured at the end of each cycle. Baseline corrected raw fluorescence (ΔR) in the Agilent AriaMX software 1.71 (Agilent) was used for setting the fluorescence thresholds. Signals exceeding this threshold with cycle threshold (Ct) value of 45 or below were considered positive.

Interpretation of RT-PCR results of the air samples were blinded to the clinical information of the participants. Analysis results of the air samples were subsequently sent to a clinician at Hvidovre Hospital who combined the data with the clinical information.

### Statistical analysis

Baseline characteristics are presented as numbers with percentages or medians with an interquartile range (IQR).

To assess any association of positive air samples between groups of participants, generalized linear mixed-effects models were performed. Fixed effects in the models were exposures of interest and included symptom duration (≤ four or > four days from symptom onset to air sampling), symptoms at time of air sampling (yes or no), variant of SARS-CoV-2 (wildtype or B.1.1.7) and method of air sampling (singing/loud talking or exhaling air), respectively. These exposures were chosen based on previous literature, as it was hypothesized that they could influence the viral load and hence could affect the amount of SARS-CoV-2 in exhaled breath^11–14^. Random within person intercept was used to account for baseline variability between participants in all four models. Furthermore, random intercept of each day of air sampling was included in the model of method of air sampling to account for variability between days.

Difference in Ct values between subgroups were assessed with linear mixed-effects models. Fixed effects in the models corresponded to the exposures of interest in our generalized linear mixed-effects models, and random intercept of each participant was also included. Additionally, random intercept of each day of air sampling was included in the model.

Estimates of association were presented as odds ratios (OR) with 95% confidence interval (95% CI). Goodness-of-fit for generalized linear mixed models was evaluated with Hosmer–Lemeshow test.

*P* values < 0.05 were considered statistically significant. Data analysis were performed using R version 3.6.0 (R Foundation for Statistical Computing, Vienna, Austria).

## Results

**Figure 1:**
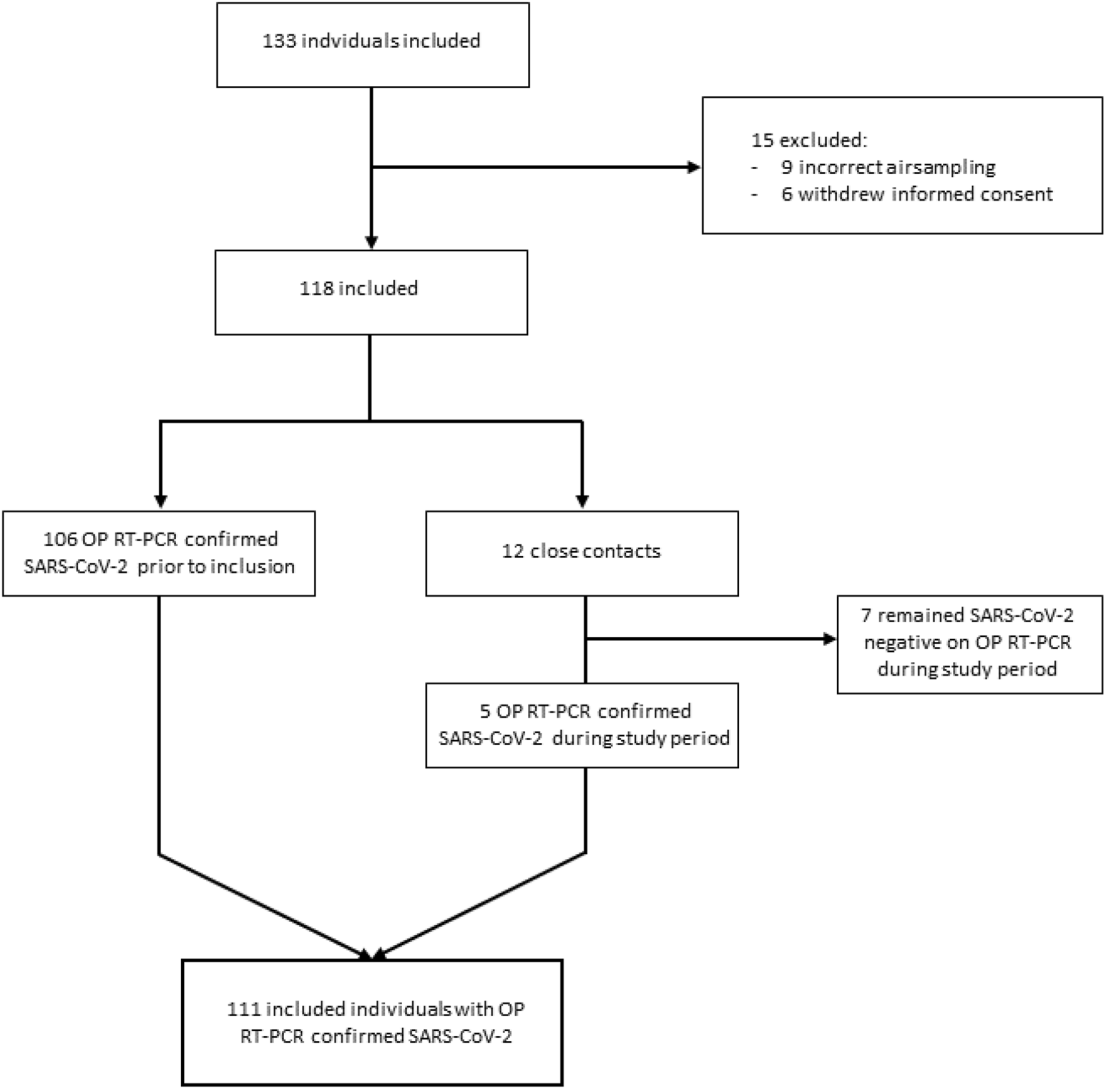
Flow chart of enrollment of participants. *OP* oropharyngeal, *RT-PCR* reverse transcriptase polymerase chain reaction, *SARS-CoV-2* severe acute respiratory coronavirus 2.

We recruited 133 individuals of which 22 were excluded (Fig. 1) resulting in 111 included subjects with RT-PCR confirmed SARS-CoV-2. Clinical characteristics of the included participants are shown in Table 1.

**Table 1:** Characteristics of subjects with confirmed SARS-CoV-2 infection.

Characteristics	Overall (n = 111)
**Sex**	
Female, n (%)	66 (59.5)
Male, n (%)	45 (40.5)
Age, median [IQR]	40 [29, 54]
**Comorbidity**	
Yes, n (%)	33 (30.0)
No, n (%)	77 (70.0)
**SARS-CoV-2 confirmed prior to inclusion**	
Yes, n (%)	106 (95.5)
No, n (%)	5 (4.5)
**Symptoms at inclusion**	
Yes, n (%)	100 (90.1)
No, n (%)	11 (9.9)
**Symptom duration at inclusion, days, median [IQR]**	3 [2, 5]
Missing, n (%)	2 (2)
**Symptoms at any time**	
Yes, n (%)	105 (95.5)
No, n (%)	5 (4.5)
Missing, n (%)	1
**SARS-CoV-2 subtype**	
Wildtype, n (%)	18 (19.8)
B.1.1.7, n (%)	73 (80.2)
Missing	20
**Complete air sampling (5 consecutive days)**	
Yes, n (%)	102 (91.9)
No, n (%)	9 (8.1)
**Positive air sample at any time**	
Yes, n (%)	52 (46.8)
No, n (%)	59 (53.2)
**Days from symptom onset to first positive air sample, median [IQR]**	3 [2, 4]

*n* number, *IQR* interquartile range, *SARS-CoV-2* severe acute respiratory coronavirus 2.

Of the 111 individuals in the study, 52 (46.8%) had at least one positive air sample. A total of 665 air samples were obtained, 536 during singing/loud talking and 129 during exhalation, from the RT-PCR confirmed individuals (Table 2). Of the 665 air samples, 129 (19.4%) were found positive for SARS-CoV-2.

**Table 2:** Number of air samples (positive/negative) stratified by exposures.

Exposure	Positive air samples, n (%)	Negative air samples, n (%)	Total air samples, n
All air samples^a^	129 (19.4)	536 (80.6)	665
**Symptoms**^**b**^			
Symptomatic patients	95 (21.1)	355 (78.9)	450
Asymptomatic patients	6 (7.2)	77 (92.8)	83
**Duration of symptoms**^**b**^			
Symptom duration ≤ 4 days	52 (26.8)	142 (73.2)	194
Symptom duration > 4 days	44 (14.5)	260 (85.5)	304
**Variant of SARS-CoV-2**^**b**^			
Wildtype	18 (20.7)	69 (79.3)	87
B.1.1.7	78 (22.0)	276 (78.0)	354
**Method of air sampling**			
Singing/loud talking	101 (18.8)	435 (81.2)	536
Exhaling	28 (21.7)	101 (78.3)	129

*n* number, *SARS-CoV-2* severe acute respiratory coronavirus 2.

^a^Obtained through singing/loud talking and exhaling.

^b^Obtained through singing/loud talking.

### Subgroup analyses

**Figure 2:**
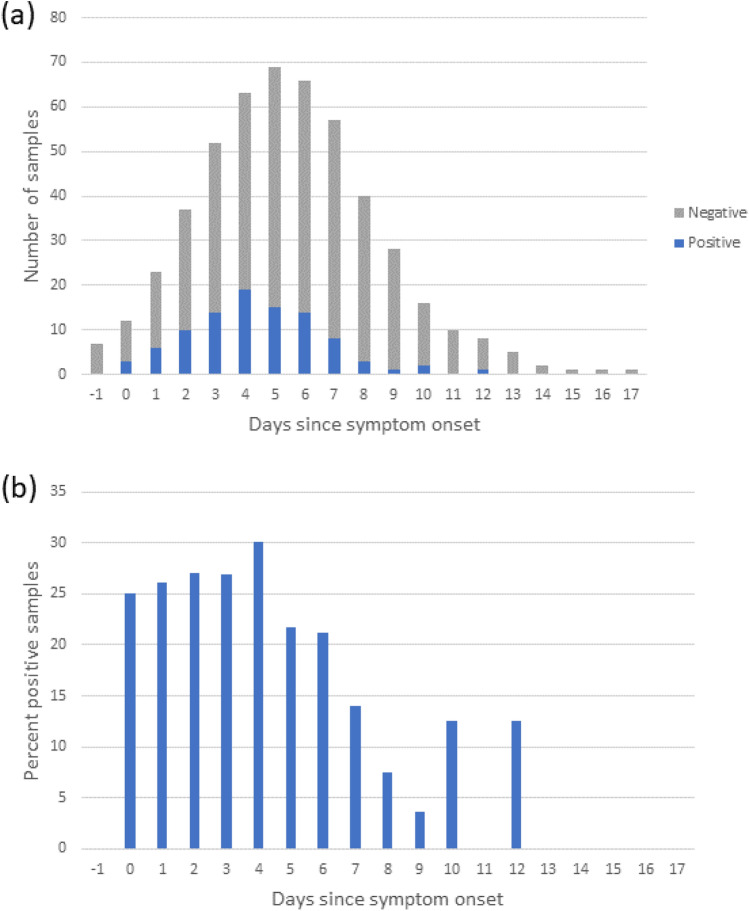
(**a**) Total number and number of positive air samples per day from symptom onset, and (**b**) percent positive air samples per day from symptom onset obtained through singing/loud talking from confirmed COVID-19 patients.

**Figure 3:**
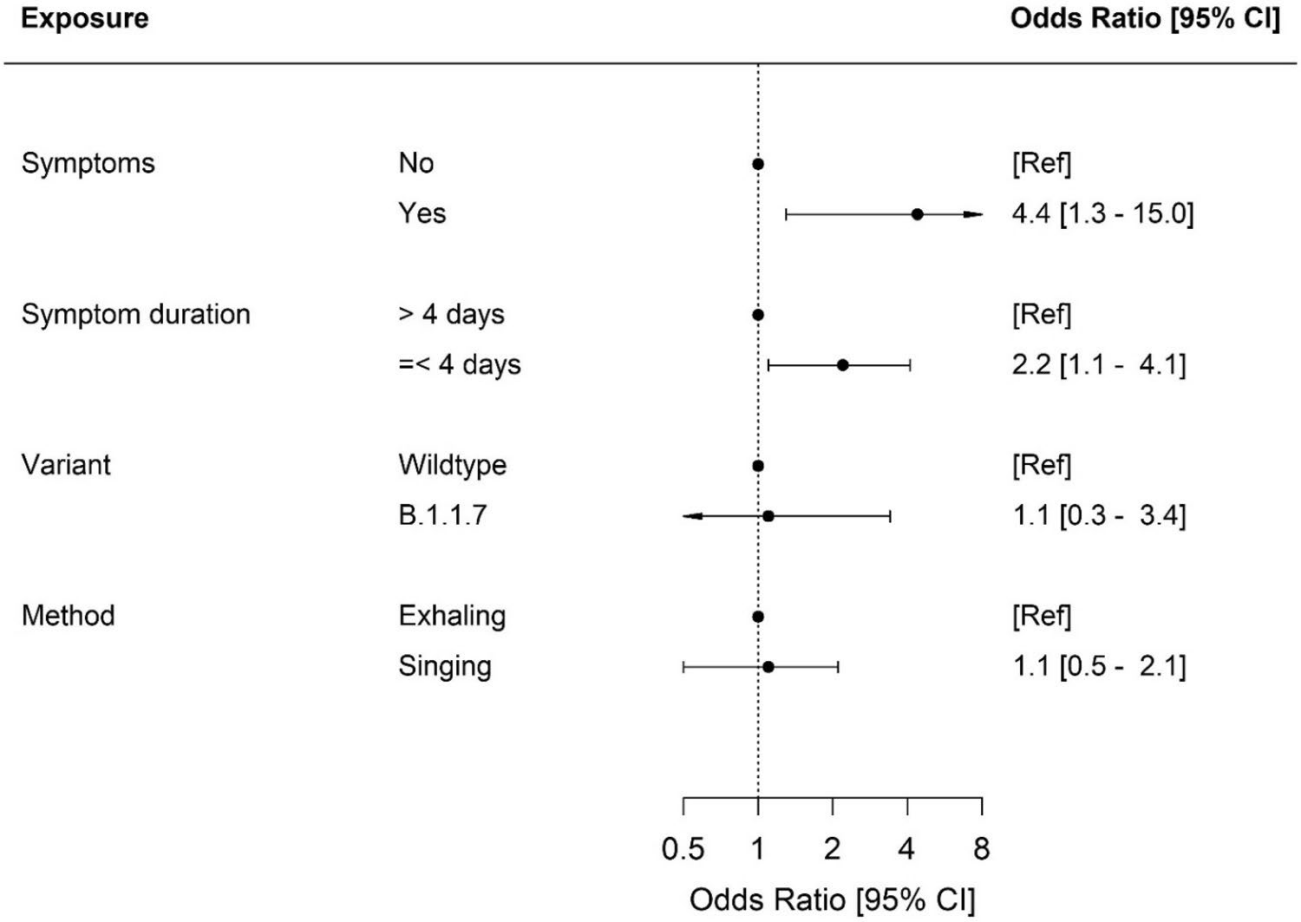
Generalized linear mixed model analyses of having a positive air sample stratified by exposures presented as OR. *CI* confidence interval, *OR* odds ratio, *ref* reference.

Figure 2 and Supplementary Fig. S2 show the distribution of air samples obtained through singing/loud talking and through exhaling according to days from symptom onset, respectively. Generalized linear mixed-effects model estimates of having a positive air sample stratified by different exposures are demonstrated in Fig. 3. Linear mixed-effect models for Ct values stratified by different exposures are presented in Table 3. Akaike Information Criterion of the statistical models are found in Supplementary Tables S1 and S2.

**Table 3:** Linear mixed-effect model analyses for Ct values stratified by exposures.

Exposure	Mean Ct value [95% CI]	Mean difference of Ct value [95% CI]	P value
Symptoms*			
Asymptomatic patients	37.17 [34.75; 39.60]	Ref	
Symptomatic patients	35.77 [35.16; 36.38]	− 1.40 [− 3.90; 1.09]	0.267
Duration of symptoms*			
Symptom duration > 4 days	36.74 [35.88; 37.59]	Ref	
Symptom duration ≤ 4 days	34.99 [34.21; 35.78]	− 1.74 [− 2.90; − 0.57]	0.004
Variant of SARS-CoV-2*			
Wildtype	35.80 [34.43; 37.18]	Ref	
B.1.1.7	35.71 [35.05; 36.36]	− 0.10 [− 1.63; 1.42]	0.894
**Method of air sampling**			
Exhaling	35.54 [34.22; 36.86]	Ref	
Singing/loud talking	35.92 [35.02; 36.83]	0.38 [− 0.90; 1.67]	0.545

*Ct* cycle threshold, *CI* confidence interval, *IQR* interquartile range, *Ref* reference, *SARS-CoV-2* severe acute respiratory coronavirus 2.

*Samples obtained through singing/loud talking.

Individuals with symptoms at the time of air sampling had significantly higher odds of having a positive air sample compared to asymptomatic individuals (OR, 4.4; p = 0.017). The mean difference of Ct values between symptomatic and asymptomatic participants was not significant (P = 0.267).

The odds of having a positive air sample among participants with a short duration of symptoms (≤ four days) compared to a longer symptom duration (> four days) were significantly higher (OR, 2.2, p = 0.017). Cycle threshold values of air samples from participants with a shorter duration of symptoms were significantly lower compared to samples obtained from participants with a longer symptom duration (p = 0.004).

We found no difference in the OR of having a positive sample, nor in the Ct values between groups of SARS-CoV-2 variant (B.1.1.7 or wildtype) or method of air sampling (singing/loud talking or exhaling).

When evaluating assumptions of linear mixed-effect models, some models did not have normally distributed residuals. Therefore, secondary models excluding the measurements deviating from the normal assumption were fitted for comparison. None of the secondary models led to different interpretation of the results, therefore results from the original models are presented.

## Discussion

This is the largest study to date to evaluate SARS-CoV-2 in exhaled breath over five consecutive days with more than 100 confirmed SARS-CoV-2 infected individuals providing more than 650 air samples. In this proof-of-concept study, we utilized an electrostatic air sampler for detection of SARS-CoV-2 in exhaled breath and demonstrated that detection rates were highest among symptomatic individuals and early in the disease course.

Different studies have investigated exhaled breath as a biological matrix in the diagnosis of SARS-CoV-2^15–21^. The diversity of devices and techniques is wide which is reflected in the varying detection rates (11.1%-70.0%) of these studies. The variation in the detection rates of SARS-CoV-2, influenced by different air sampling techniques, is further supported in a review by Borges et al., comparing 25 papers reporting on air sampling and detection methods for SARS-CoV-2 in indoor environments^21^. Among these studies, 10 did not detect SARS-CoV-2 in air while 15 detected SARS-CoV-2 with sensitivities varying between 2 and 100%. Two studies reported sensitivities above 65%, one study using a solid impactor and another using a filter-based method^21–23^. However, lower sensitivities have also been reported in other studies using the same methods^21^. The large variation in sensitivity likely reflects different study design and methological approaches, e.g. sampling time and airflow rate. An overall detection rate in this study of 19.4% was found when including all analyzed air samples, which were obtained from one day prior to symptom onset and up to 17 days after symptom onset. The detection rate increased to 26.8% in earlier stages of the disease, i.e. in samples obtained ≤ 4 days from symptom onset. When taking individual variability of each participants into account, the odds of having a positive air sample were 2.2 times higher in air samples obtained ≤ 4 days from symptom onset compared to > four days. This corresponds to the significantly lower Ct value observed in these samples compared to Ct values in samples obtained in later stages of the disease. Previous studies have demonstrated that viral load in oro-/nasopharyngeal swabs peak on or soon after the day of symptom onset and then decline, which is in accordance with our results^12,13,24^. Other papers using air sampling for detection of SARS-CoV-2 have also reported higher detection rates and/or lower Ct values with shorter symptom duration^16,17,20^.

Participants with symptoms at the time of air sampling had 4.4 higher odds of having a SARS-CoV-2 positive air sample compared to asymptomatic participants. Previous studies evaluating exhaled air samples from COVID-19 patients only report on symptomatic patients, thus they are not directly comparable with our results. Although there was a higher proportion of positive samples from symptomatic participants, no difference in Ct value was found in symptomatic compared to asymptomatic participants in this study. Knowledge on viral load in a- and presymptomatic SARS-CoV-2-infected individuals is scarce, but a few studies suggested that viral load in naso-/oropharyngeal swabs from a- or presymptomatic individuals did not differ from viral load in symptomatic individuals^25^. In this study, only six (7.2%) air samples from asymptomatic individuals were positive, providing a wide IQR for Ct value reflecting a too small sample size of this group. Therefore, this result should be interpreted with caution.

A more transmittable lineage of SARS-CoV-2 (Alpha or B.1.1.7) was rapidly spread in December 2020 in the UK and later to other countries^26,27^. A study reported lower Ct values (i.e. higher viral load) in upper respiratory material from patients infected with B.1.1.7 compared to those infected with previous lineages^14^. No significant difference was found in neither OR of positive samples nor Ct values between air samples taken from B.1.1.7-infected individuals compared with wildtype-infected individuals.

The strength of this study is the large sample size and large number of air samples. Furthermore, our findings are in accordance with the literature, reporting higher detection rates in earlier disease stages. Despite the potential observed using air sampling for detection of SARS-CoV-2 in the early stage of the infection, our results suggest that there is still room for optimization of the method, as our overall detection rate was relatively low compared to other studies on air sampling. This could partly be explained by the fact that RT-PCR was performed on the air samples targeting only the E gene. Ryan et al. reported that the detection rate of SARS-CoV-2 increased from 68% to 93.5% using four gene targets (S/E/N/Orf1ab) instead of two targets (E and S)^15^. This indicates that analyzing several genes could increase the overall detection rate. Another approach to improve the sensitivity, is to increase the flow-rate of the AeroCollect to increase viral particle capture. A major limitation of this study is the absence of specificity data, due to the lack of a control group. With the aim of becoming relevant in a clinical setting, it is crucial that future studies include a healthy control group in order to evaluate the specificity of this detection method.

Taking the low sensitivity into account, the present study does not suggest air sampling as an alternative diagnostic method to standard oro-/nasopharyngeal swab for SARS-CoV-2 detection. Moreover, this method can not compete with the most sensitive rapid antigen tests for SARS-CoV-2^28^. However, with further optimization of air sampling, this method could play a role in population-based monitoring of SARS-CoV-2 as an early warning system. Furthermore, taking the short sampling time and non-invasive route of sampling into account, this method could also play a role as an alternative diagnostic tool when a standard oro-/nasopharyngeal swab is not possible e.g. among infants, small children and patients suffering from dementia. Lastly, the technology is easy to use and thus suitable for self-sampling. It enables direct PCR analysis without any time-consuming purification steps prior to PCR analysis.

In conclusion, in this study on detecting SARS-CoV-2 in human exhaled breath we demonstrate that the odds of having a positive air sample were significantly higher in earlier disease stages and among symptomatic individuals compared to asymptomatic individuals. For this method to be relevant in a clinical setting, future studies aiming to increase the detection rate are warranted.

## Supplementary Information

Supplementary Information.

## Data Availability

The datasets generated during and/or analysed during the current study are available from the corresponding author on reasonable request.

## References

[1] Koskinen, A. *et al.* Complications of COVID-19 Nasopharyngeal Swab Test. *JAMA Otolaryngol. Head Neck Surg.* **147**, 672–674 (2021). 10.1001/jamaoto.2021.0715 PMC8085764 33914064

[2] Föh B (2021). Complications of nasal and pharyngeal swabs: a relevant challenge of the COVID-19 pandemic?. Eur. Respir. J..

[3] Shenoy S (2021). SARS-CoV-2 (COVID-19), viral load and clinical outcomes; lessons learned one year into the pandemic: A systematic review. World J. Crit. Care Med..

[4] Tang S (2020). Aerosol transmission of SARS-CoV-2? Evidence, prevention and control. Environ. Int..

[5] Morawska L, Cao J (2020). Airborne transmission of SARS-CoV-2: The world should face the reality. Environ. Int..

[6] van Doremalen, N. *et al.* Aerosol and Surface Stability of SARS-CoV-2 as Compared with SARS-CoV-1. *N. Engl. J. Med.* NEJMc2004973 (2020). 10.1056/NEJMc2004973. 10.1056/NEJMc2004973 PMC7121658 32182409

[7] Boklund, A. *et al.* SARS-CoV-2 in Danish Mink Farms: Course of the Epidemic and a descriptive analysis of the outbreaks in 2020. *Anim. Open Access J. MDPI* **11**, (2021). 10.3390/ani11010164 PMC7828158 33445704

[8] Alsved M (2020). Exhaled respiratory particles during singing and talking. Aerosol Sci. Technol..

[9] Mürbe D, Kriegel M, Lange J, Rotheudt H, Fleischer M (2021). Aerosol emission in professional singing of classical music. Sci. Rep..

[10] Asadi, S. *et al.* Aerosol emission and superemission during human speech increase with voice loudness. *Sci. Rep.* **9**, (2019). 10.1038/s41598-019-38808-z PMC6382806 30787335

[11] Chung E (2021). Comparison of symptoms and RNA levels in children and adults with SARS-CoV-2 infection in the community setting. JAMA Pediatr..

[12] He X (2020). Temporal dynamics in viral shedding and transmissibility of COVID-19. Nat. Med..

[13] Wölfel R (2020). Virological assessment of hospitalized patients with COVID-2019. Nature.

[14] Challen R (2021). Risk of mortality in patients infected with SARS-CoV-2 variant of concern 202012/1: Matched cohort study. The BMJ.

[15] Ryan, D. J. *et al.* Use of exhaled breath condensate (EBC) in the diagnosis of SARS-COV-2 (COVID-19). *Thorax* thoraxjnl-2020–215705 (2020). 10.1136/thoraxjnl-2020-215705. 10.1136/thoraxjnl-2020-215705 33097604

[16] Sawano, M., Takeshita, K., Ohno, H. & Oka, H. RT-PCR diagnosis of COVID-19 from exhaled breath condensate: A clinical study. *J. Breath Res.* **15**, (2021). 10.1088/1752-7163/ac0414 34020435

[17] Ma J (2021). Coronavirus disease 2019 patients in earlier stages exhaled millions of severe acute respiratory syndrome coronavirus 2 per hour. Clin. Infect. Dis..

[18] Zhou L (2021). Breath-, air- and surface-borne SARS-CoV-2 in hospitals. J. Aerosol Sci..

[19] Li, X. *et al.* Detecting SARS-CoV-2 in the breath of COVID-19 patients. *Front. Med.* **8**, (2021). 10.3389/fmed.2021.604392 PMC8010128 33816516

[20] Malik, M., Kunze, A.-C., Bahmer, T., Herget-Rosenthal, S. & Kunze, T. SARS-CoV-2: viral loads of exhaled breath and oronasopharyngeal specimens in hospitalized patients with COVID-19. *Int. J. Infect. Dis. IJID Off. Publ. Int. Soc. Infect. Dis.* **110**, 105–110 (2021). 10.1016/j.ijid.2021.07.012 PMC8260556 34242768

[21] Borges JT, Nakada LYK, Maniero MG, Guimarães JR (2021). SARS-CoV-2: A systematic review of indoor air sampling for virus detection. Environ. Sci. Pollut. Res. Int..

[22] Chia PY (2020). Detection of air and surface contamination by SARS-CoV-2 in hospital rooms of infected patients. Nat. Commun..

[23] Razzini K (2020). SARS-CoV-2 RNA detection in the air and on surfaces in the COVID-19 ward of a hospital in Milan Italy. Sci. Total Environ..

[24] Zou L (2020). SARS-CoV-2 viral load in upper respiratory specimens of infected patients. N. Engl. J. Med..

[25] Byrne AW (2020). Inferred duration of infectious period of SARS-CoV-2: rapid scoping review and analysis of available evidence for asymptomatic and symptomatic COVID-19 cases. BMJ Open.

[26] Davies, N. G. *et al.* Estimated transmissibility and impact of SARS-CoV-2 lineage B.1.1.7 in England. *Science* **372**, eabg3055 (2021). 10.1126/science.abg3055 PMC8128288 33658326

[27] Bager P (2021). Risk of hospitalisation associated with infection with SARS-CoV-2 lineage B.1.1.7 in Denmark: An observational cohort study. Lancet Infect. Dis..

[28] Wagenhäuser I (2021). Clinical performance evaluation of SARS-CoV-2 rapid antigen testing in point of care usage in comparison to RT-qPCR. EBioMedicine.

