# Supplementary material for: Detection of SARS-CoV-2 in exhaled breath from non-hospitalized COVID-19-infected individuals
Source: Sci Rep. 2022 Jul 1;12:11151. doi: 10.1038/s41598-022-15243-1 (PMC9247943; doi:10.1038/s41598-022-15243-1)
Supplement: Supplementary file 1 — Supplementary Information. [file 41598_2022_15243_MOESM1_ESM.docx]

**Detection of SARS-CoV-2 in exhaled breath from non-hospitalized COVID-19-infected individuals**

Cæcilie Leding*, MD^1^, Julia Skov, MSc^2^, Katrine Uhrbrand, PhD^2^, Jan Gorm Lisby, MD^3^, Katrine Pedersbæk Hansen, MD^1^, Thomas Benfield, MD^1,4^ and Louise Katrine Duncan, PhD^2^

^1^Center of Research & Disruption of Infectious Diseases, Department of Infectious Diseases, Copenhagen University Hospital – Amager and Hvidovre, Hvidovre, Denmark

^2^AeroCollect, FORCE Technology, Hørsholm, Denmark

^3^Department of Clinical Microbiology, Copenhagen University Hospital – Amager and Hvidovre, Hvidovre, Denmark

^4^Department of Clinical Medicine, Faculty of Health and Medical Sciences, University of Copenhagen, Copenhagen, Denmark

**Supplementary Figure S1.** Illustration of air sampling and sample elution using the AeroCollect device. (a) The AeroCollect device and disposable sample chamber. (b) Air is pulled through the sample chamber capturing virus particles within the chamber between air inlet and air outlet. (c) The sample chamber is inserted into the AeroCollect device. (d) By pressing the ON button air is pulled through the chamber and an electrostatic field is generated across the chamber. (e) Sampling from exhaled breath is performed close to the mouth. (f) The sampler is turned off by pressing the OFF button. (g) The sample chamber is removed from the AeroCollect device. (h) The sample chamber is flushed with 25µl nuclease free water. The sample is ready for direct RT-PCR analysis without any purification steps.


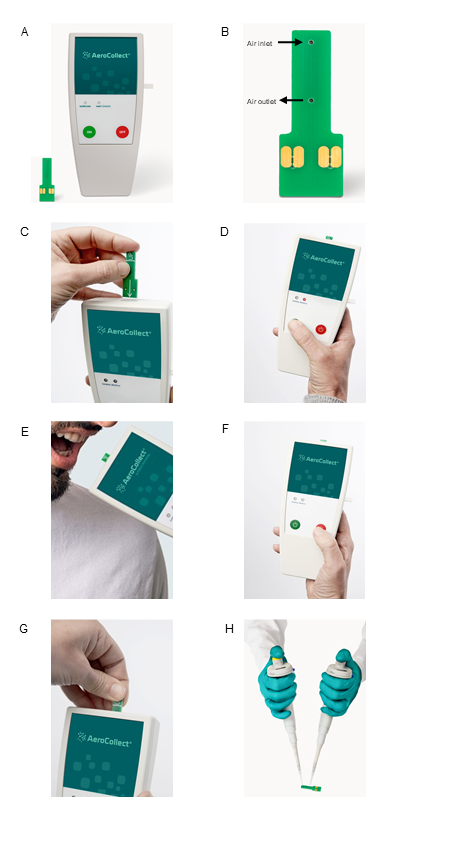


**Supplementary Figure S2.** (**a**) Total number and number of positive air samples per day from symptom onset, and (**b**) percent positive air samples per day from symptom onset obtained through exhaling from confirmed COVID-19 patients.

**
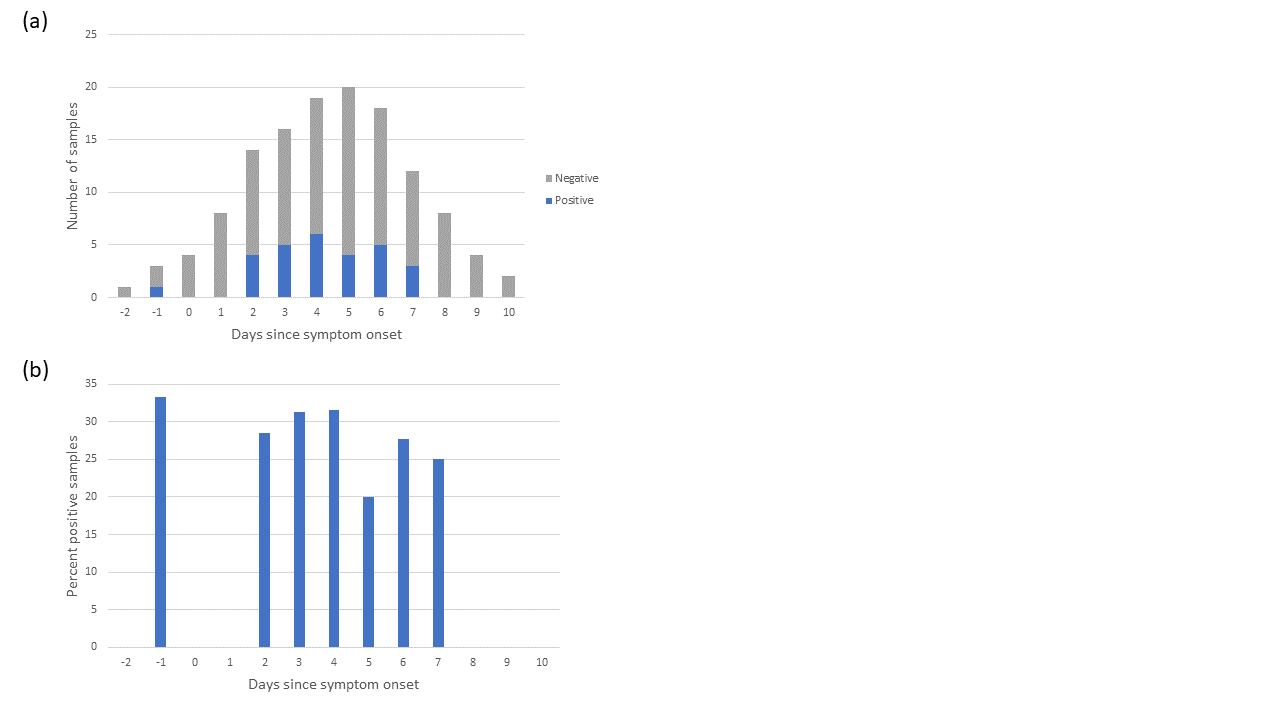
**

**Supplementary Table S1.**  Generalized linear mixed effect models for positive samples.

| Exposure | AIC |
| --- | --- |
| Symptoms yes/no | 464.3 |
| Symptoms duration | 440.0 |
| Variant of SARS-CoV-2 | 423.6 |
| Method of air sampling | 578.9 |

Abbreviation: AIC, Akaike Information Criterion; SARS-CoV-2, severe acute respiratory coronavirus 2.

**Supplementary Table S2.** Linear mixed effect models for difference in Ct values.

| Exposure | AIC |
| --- | --- |
| Symptoms yes/no | 512.3 |
| Symptoms duration | 478.2 |
| Variant of SARS-CoV-2 | 483.6 |
| Method of air sampling | 662.1 |

Abbreviation: AIC, Akaike Information Criterion; SARS-CoV-2, severe acute respiratory coronavirus 2.
